# Supplementary material for: Beyond 40 fluorescent probes for deep phenotyping of blood mononuclear cells, using spectral technology
Source: Front Immunol. 2024 Apr 2;15:1285215. doi: 10.3389/fimmu.2024.1285215 (PMC11018965; doi:10.3389/fimmu.2024.1285215)

Supplementary Figure 1

A AF647 and SparkNIR-685: Similarity Index = 0.74

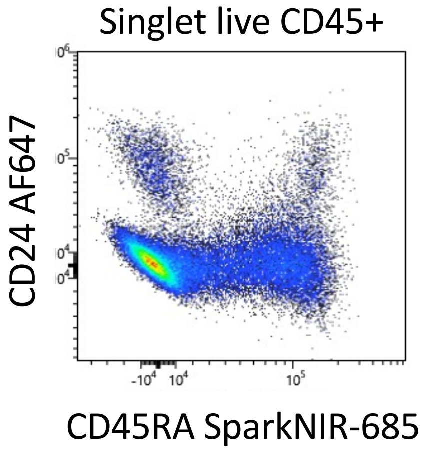

B PE-Cy5 and PE-Fire640: Similarity Index = 0.77

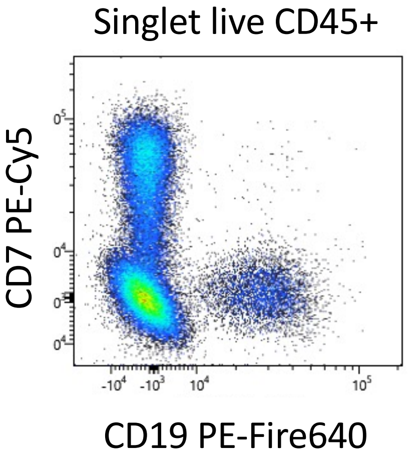

C BV510 and BV480: Similarity Index = 0.78

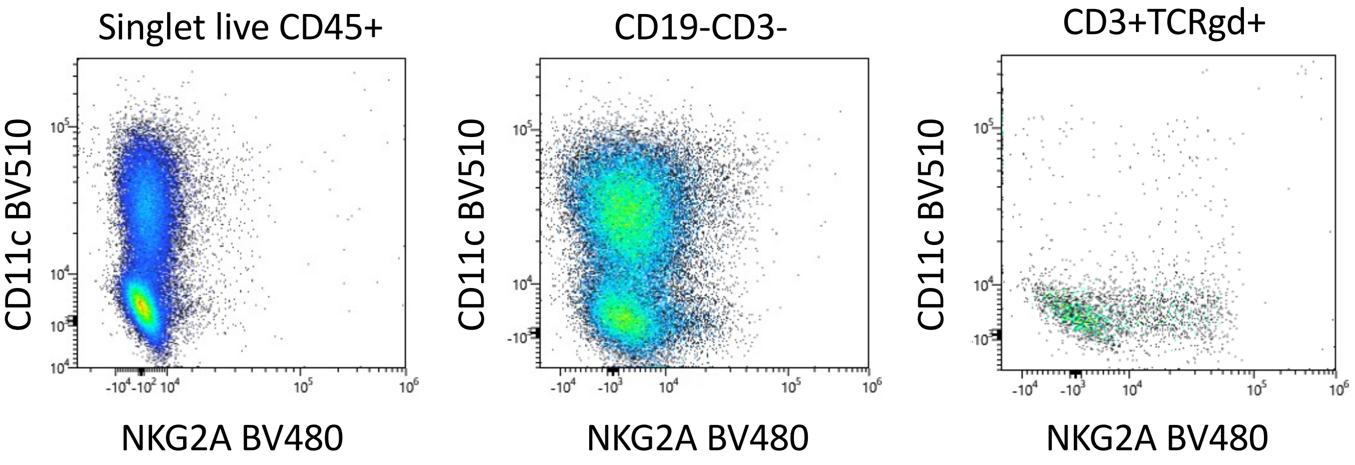

Supplement: Supplementary Figure 1 — Unmixing of dyes with high similarity index. Singlet live CD45+ populations are shown after unmixing. (A) AF647 and SparkNIR685 have a similarity index of 0.74 (B) PE-Cy5 and PE-Fire810 have a similarity index of 0.77 C. BV480 and BV510 have a similarity index of 0.78. Different populations are shown to validate the specificity of the marker NKG2A that is not visible on total singlets live CD45+ cells. [file DataSheet_1.pdf]
