# Supplementary figures and images for: Beyond 40 fluorescent probes for deep phenotyping of blood mononuclear cells, using spectral technology
Source: Front Immunol. 2024 Apr 2;15:1285215. doi: 10.3389/fimmu.2024.1285215 (PMC11018965; doi:10.3389/fimmu.2024.1285215)

# Supplementary Figure 2

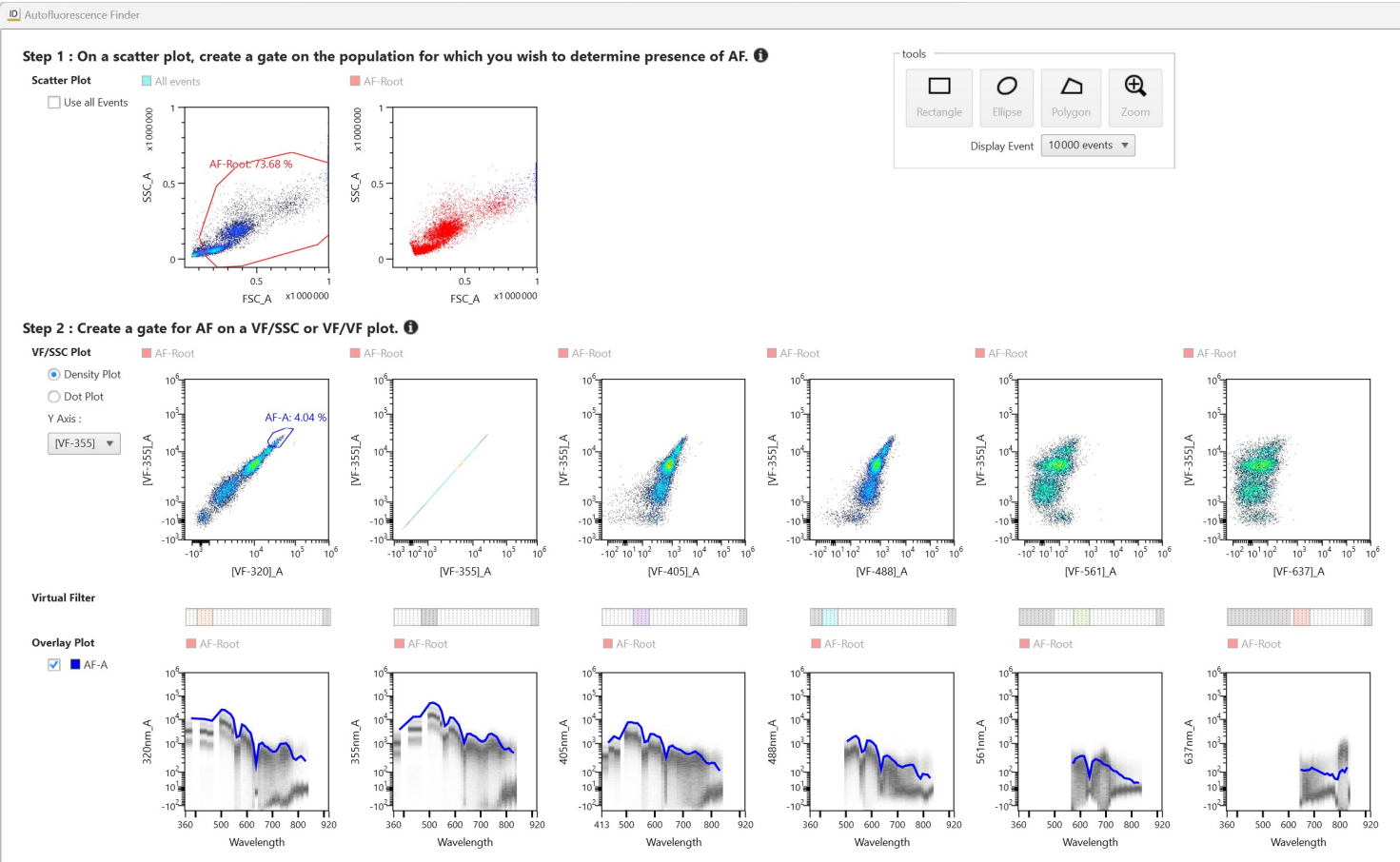

Supplement: Supplementary Figure 2 — AutoFluorescence Finder tool in ID7000 software. (A) Definition of the autofluorescent population using the Autofluorescence Finder tool of the ID7000 software. The spectral shape of the autofluorescent population is shown in blue. [file DataSheet_2.pdf]
