# Supplementary material for: Beyond 40 fluorescent probes for deep phenotyping of blood mononuclear cells, using spectral technology
Source: Front Immunol. 2024 Apr 2;15:1285215. doi: 10.3389/fimmu.2024.1285215 (PMC11018965; doi:10.3389/fimmu.2024.1285215)

Supplementary Figure 3 (1/2)

A 5 lasers + AF management / gated on singlet live CD45+ cells

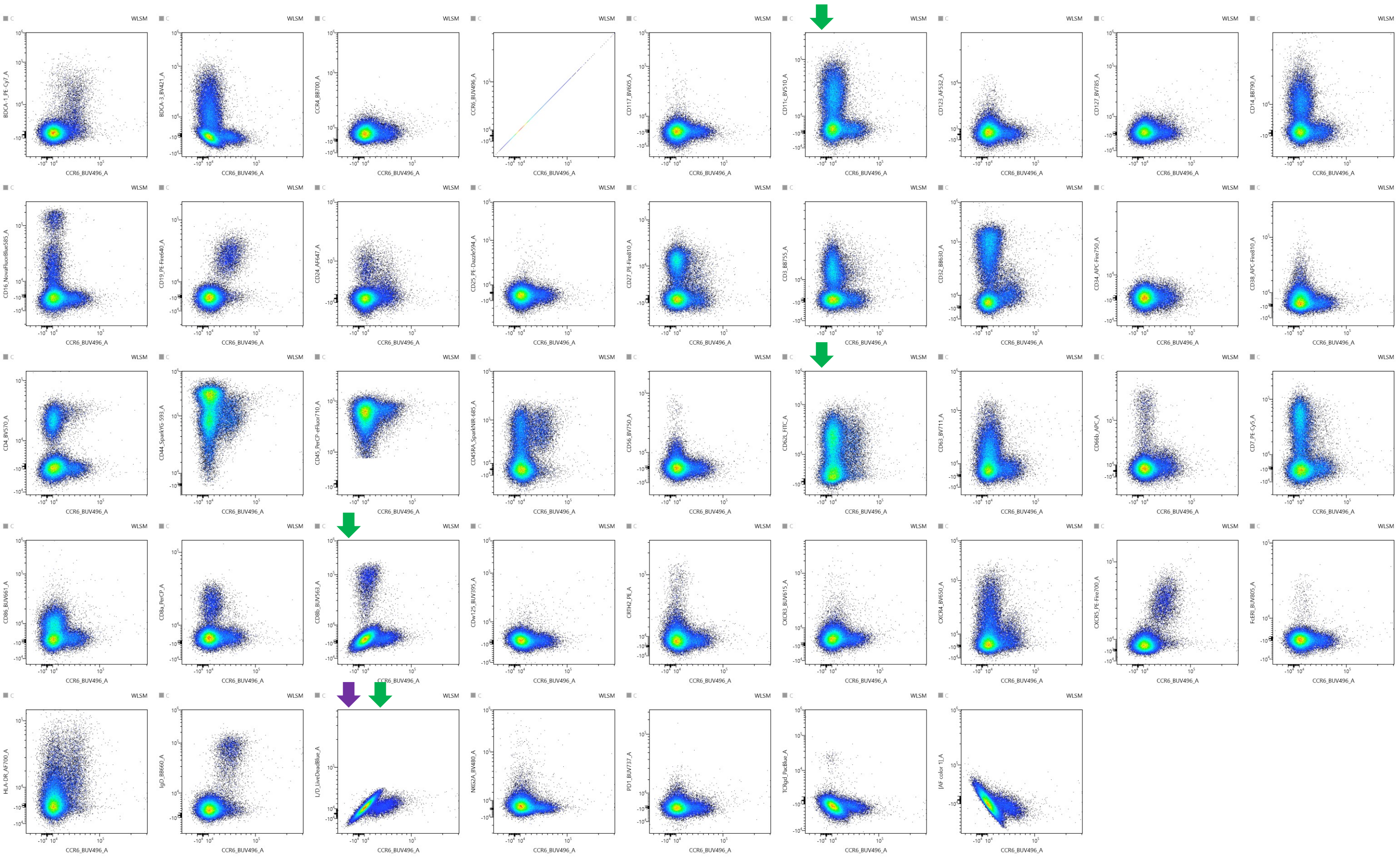

B 6 lasers + AF management / gated on singlet live CD45+ cells

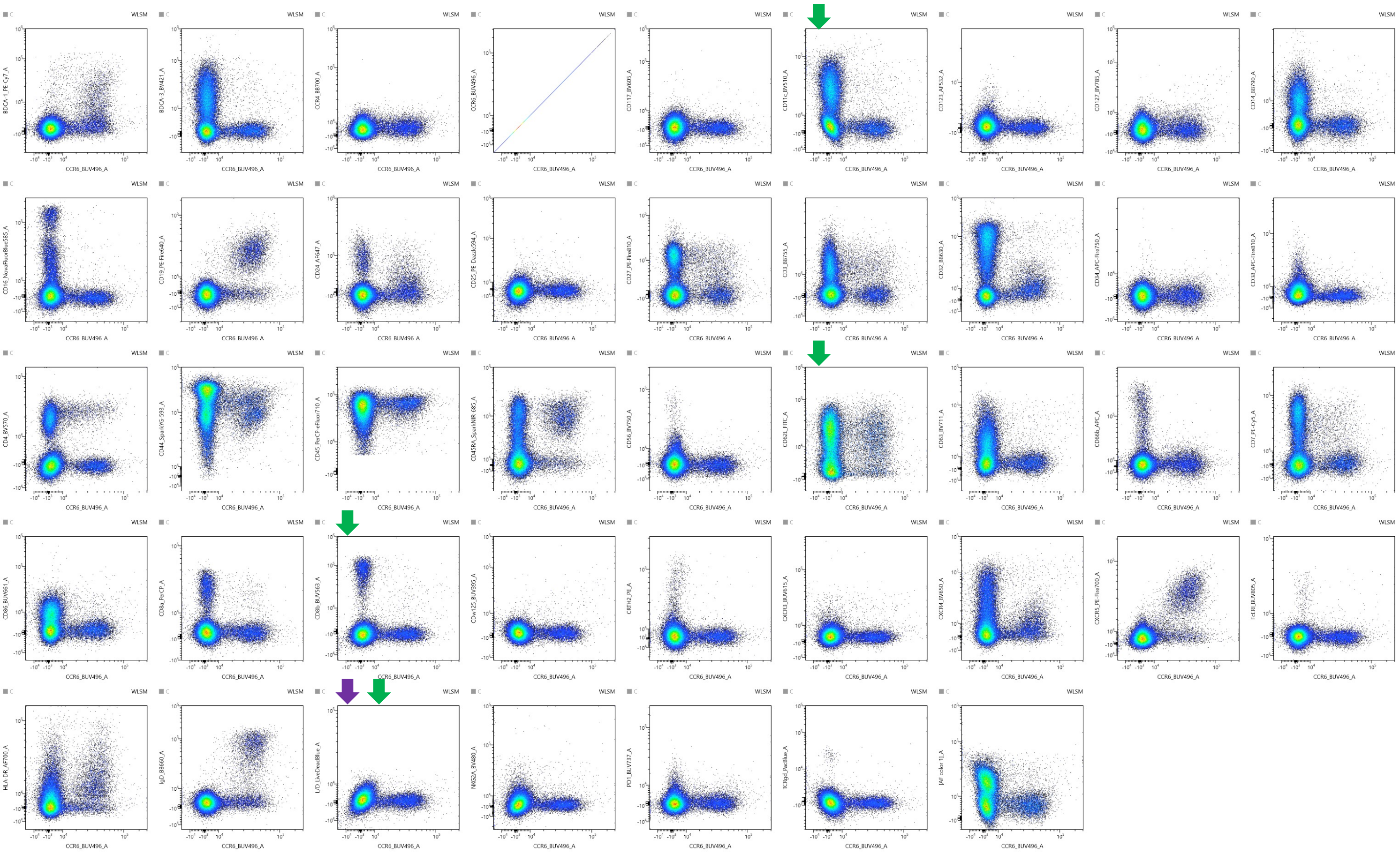

Supplementary Figure 3 (2/2)

C 5 lasers no AF management / gated on singlet live CD45+ cells

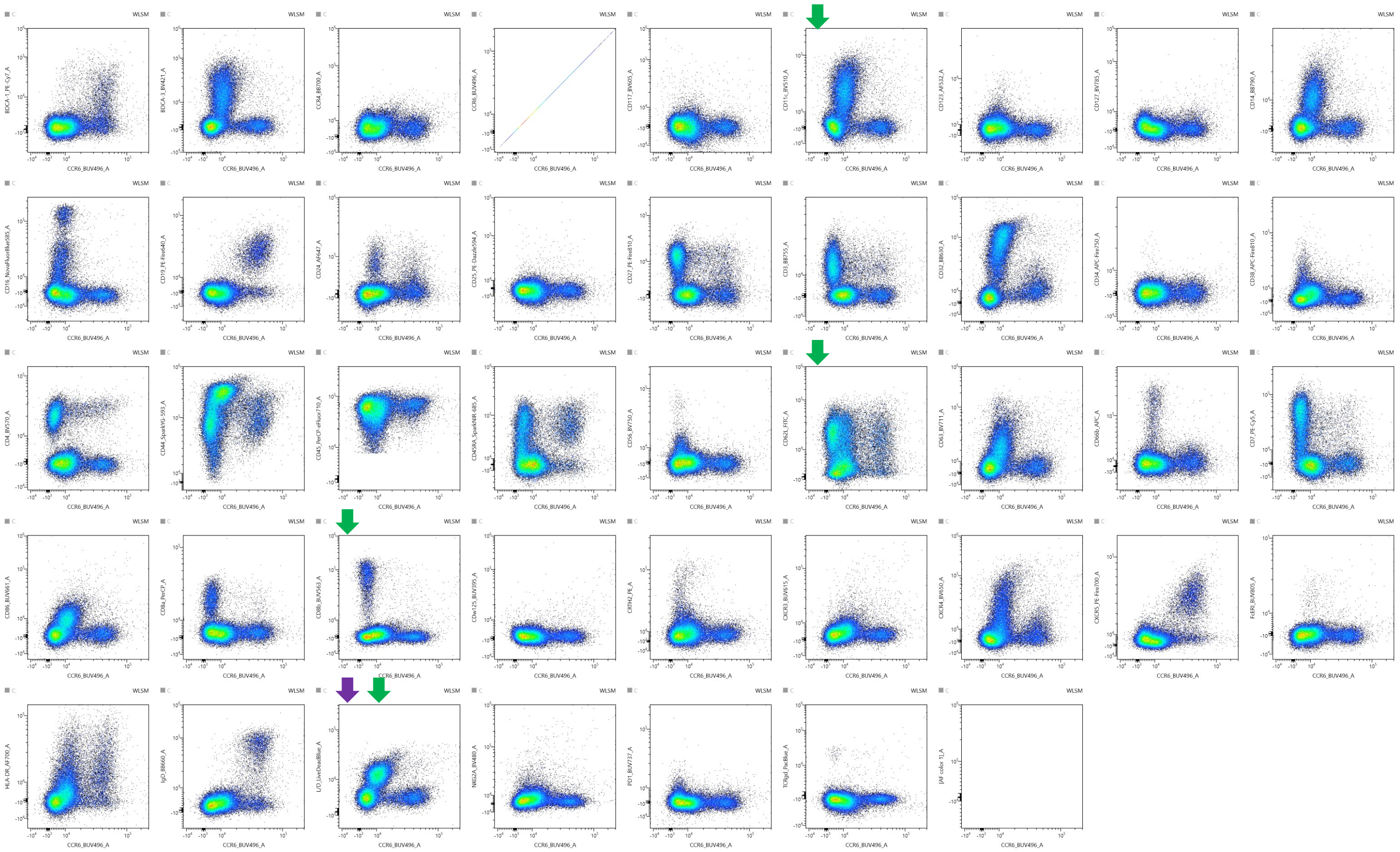

Supplement: Supplementary Figure 3 — Impact of 320nm deep-UV laser. Comparison of the signal resolution and spill over upon acquisitions of the cells with 5 lasers (A, C) or 6 lasers, including 320nm laser (B) and analysis of the generated data with AF management (A, B) or without (C). The example of the expression of BUV496 signal vs each fluorophore of the panel is shown. [file DataSheet_3.pdf]
