# Supplementary material for: Beyond 40 fluorescent probes for deep phenotyping of blood mononuclear cells, using spectral technology
Source: Front Immunol. 2024 Apr 2;15:1285215. doi: 10.3389/fimmu.2024.1285215 (PMC11018965; doi:10.3389/fimmu.2024.1285215)

Supplementary Figure 5 (1/2)

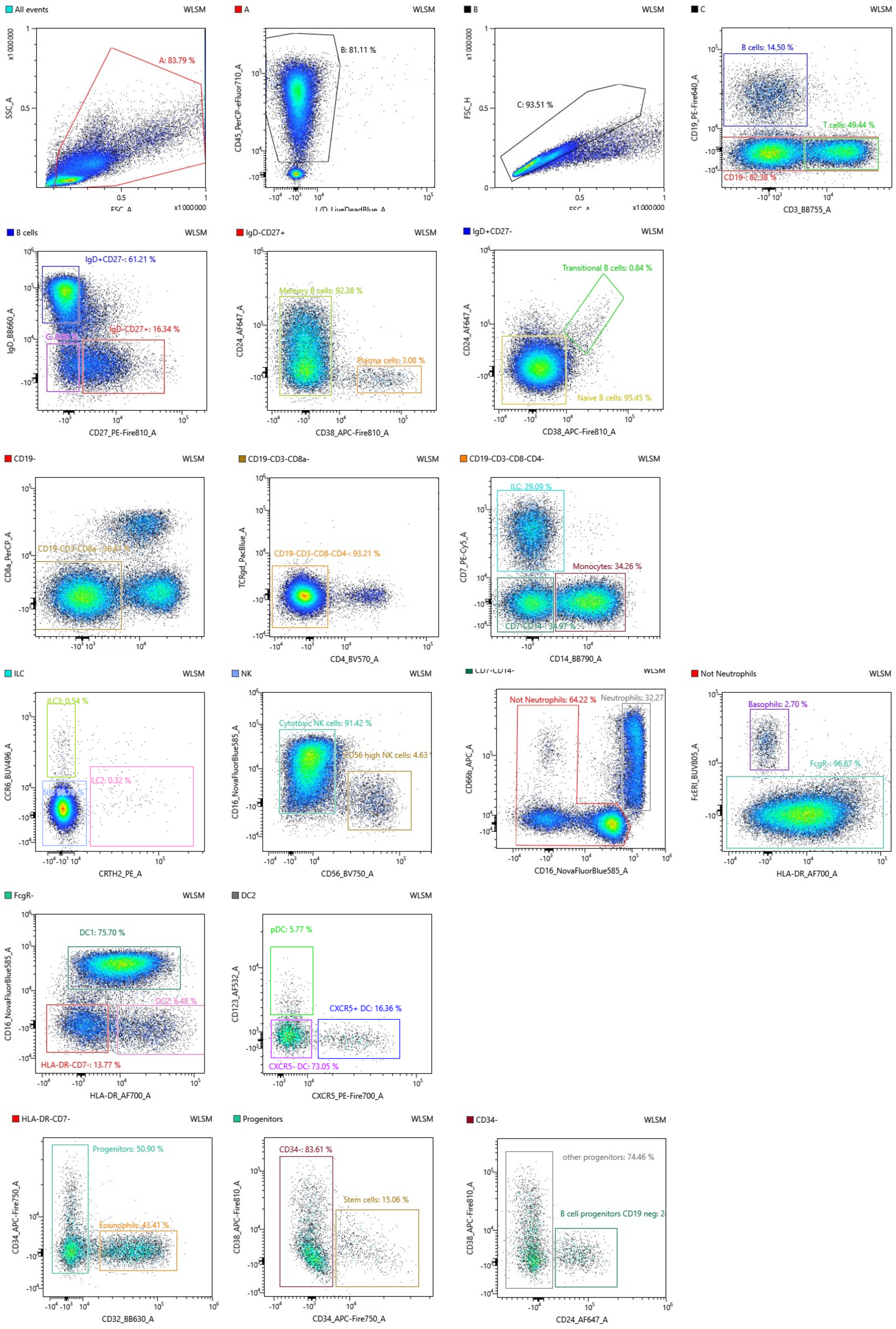

Supplementary Figure 5 (2/2)

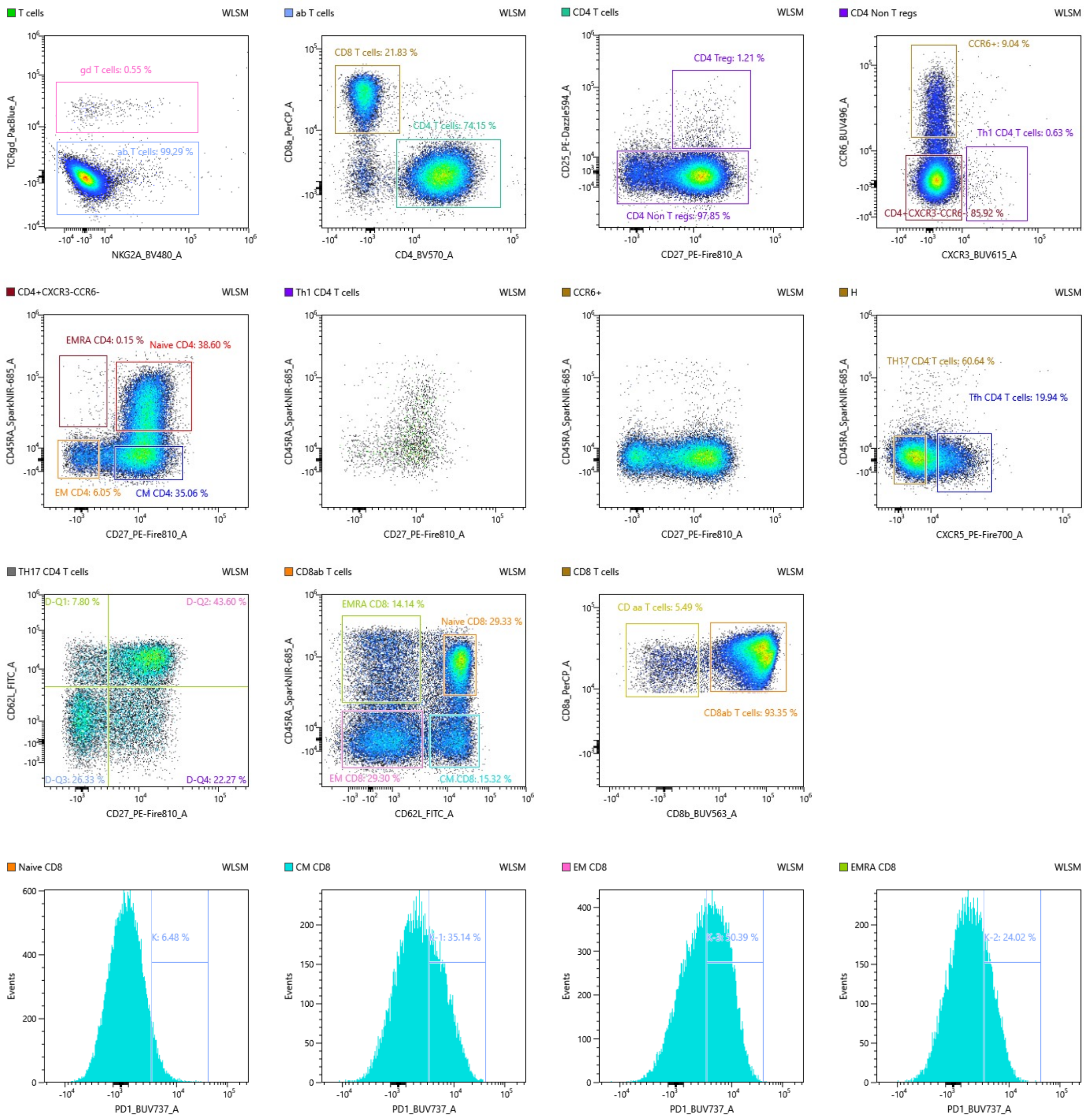

Supplement: Supplementary Figure 5 — Plots showing the total manual gating strategy. The colors of the populations shown on the Flt-SNE plots () represent the manual gated populations shown in the figure. [file DataSheet_5.pdf]
