# Supplementary material for: Beyond 40 fluorescent probes for deep phenotyping of blood mononuclear cells, using spectral technology
Source: Front Immunol. 2024 Apr 2;15:1285215. doi: 10.3389/fimmu.2024.1285215 (PMC11018965; doi:10.3389/fimmu.2024.1285215)

Supplementary Figure 4

A

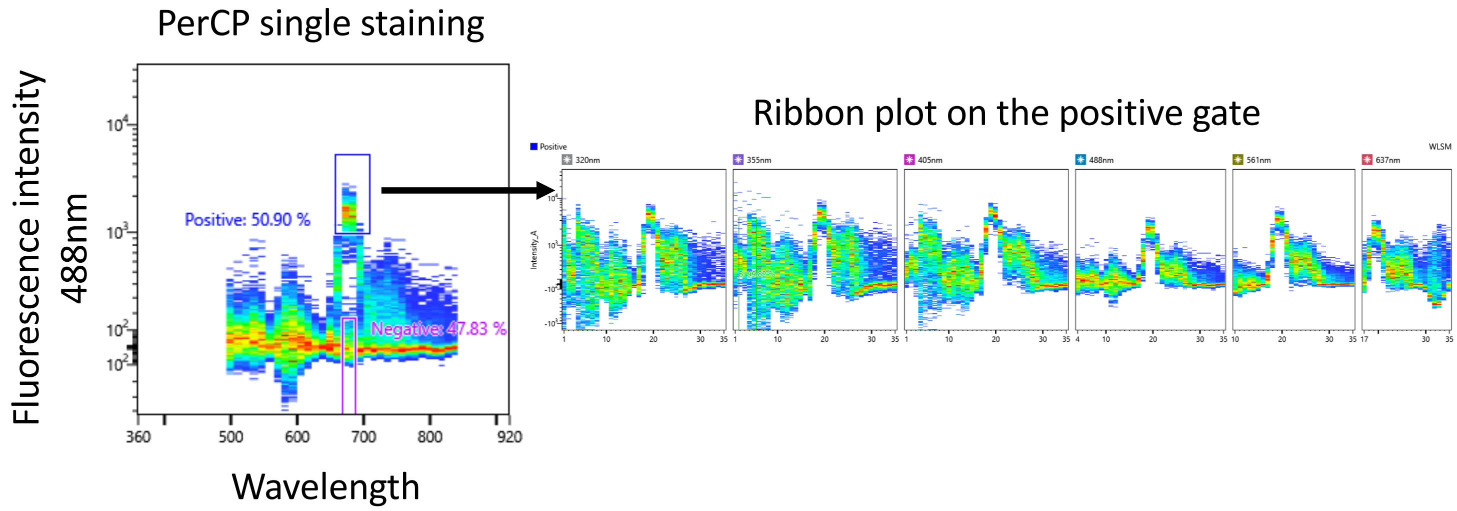

B After unmixing / gated on singlet live CD45+

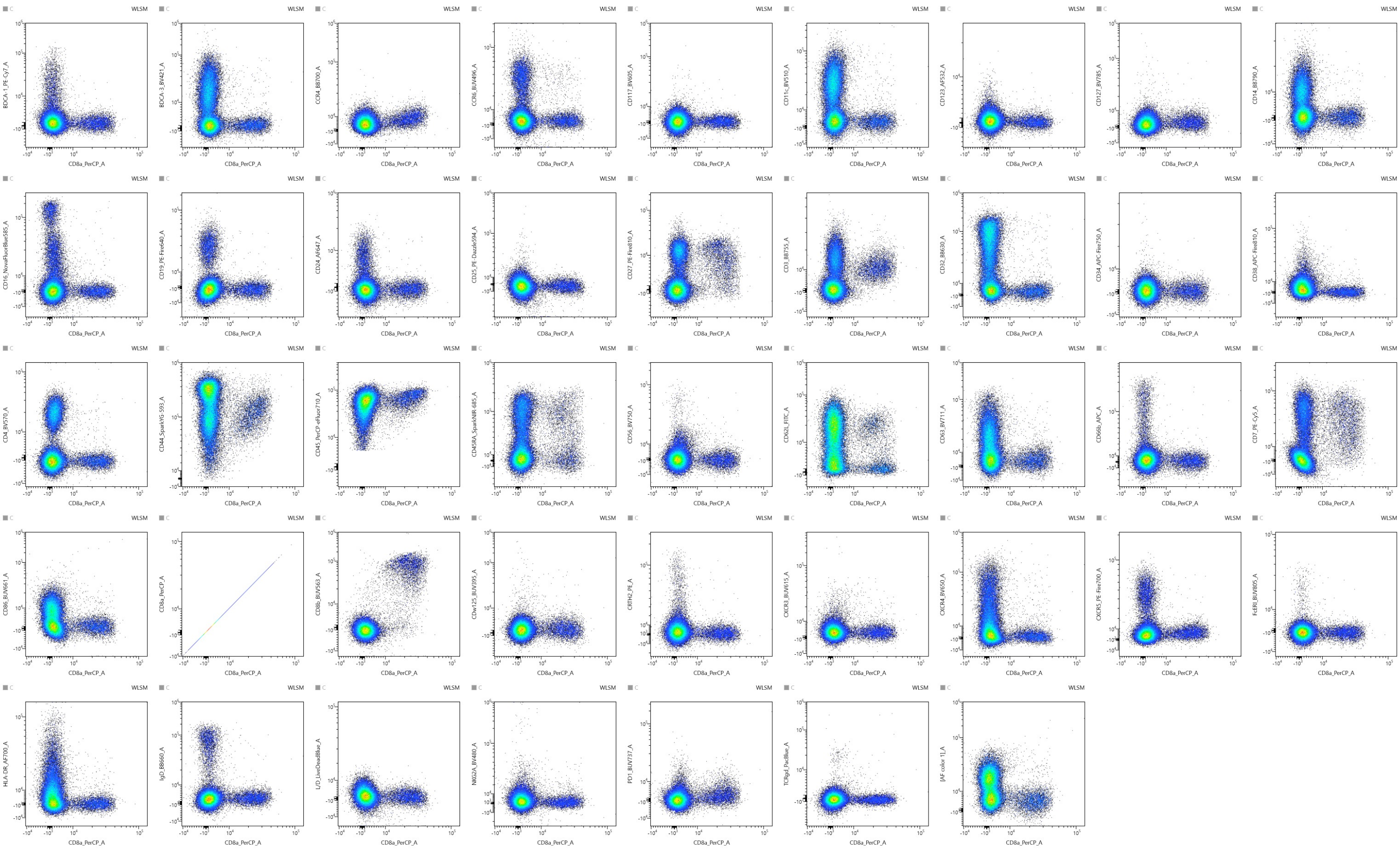

Supplement: Supplementary Figure 4 — Unmixing was validated with the NxN matrix showing all bi-parametric plots for one dye after the other. (A) Example of assignment of positive and negative population from single stained sample of PerCP (left plot). The ribbon plot shows the spectrum of the positive gate of PerCP all along the detectors of all lasers. (B) NxN matrix with all combinations is a tool to verify and validate the quality of the unmixing for each dye, one after the other, versus all the other dyes. Here is shown PerCP versus all other dyes of the panel. [file DataSheet_4.pdf]
